# Structurally Defined Potassium-Mediated Zincation of Pyridine and 4-R-Substituted Pyridines (R=Et, *i*Pr, *t*Bu, Ph, and Me_2_N) by Using Dialkyl–TMP–Zincate Bases

**DOI:** 10.1002/chem.200900549

**Published:** 2009-06-16

**Authors:** William Clegg, Ben Conway, David V Graham, Eva Hevia, Alan R Kennedy, Robert E Mulvey, Luca Russo, Dominic S Wright

**Affiliations:** [a] WestCHEM, Department of Pure and Applied Chemistry, University of StrathclydeGlasgow, G1 1XL (UK), Fax: (+44)141-552-0876; [b] School of Chemistry, Newcastle UniversityNewcastle upon Tyne, NE1 7RU (UK); [c] Chemistry Department, University of CambridgeLensfield Road, Cambridge, CB2 1EW (UK)

**Keywords:** alkali metals, metalation, potassium, pyridine, zincation

## Abstract

Two potassium–dialkyl–TMP–zincate bases [(pmdeta)K(μ-Et)(μ-tmp)Zn(Et)] (**1**) (PMDETA=*N*,*N*,*N′*,*N′′*,*N′′*-pentamethyldiethylenetriamine, TMP=2,2,6,6-tetramethylpiperidide), and [(pmdeta)K(μ-*n*Bu)(μ-tmp)Zn(*n*Bu)] (**2**), have been synthesized by a simple co-complexation procedure. Treatment of **1** with a series of substituted 4-R-pyridines (R=Me_2_N, H, Et, *i*Pr, *t*Bu, and Ph) gave 2-zincated products of the general formula [{2-Zn(Et)_2_-μ-4-R-C_5_H_3_N}_2_**⋅**2{K(pmdeta)}] (**3**–**8**, respectively) in isolated crystalline yields of 53, 16, 7, 23, 67, and 51%, respectively; the treatment of **2** with 4-*t*Bu-pyridine gave [{2-Zn(*n*Bu)_2_-μ-4-*t*Bu-C_5_H_3_N}_2_**⋅**2{K(pmdeta)}] (**9**) in an isolated crystalline yield of 58%. Single-crystal X-ray crystallographic and NMR spectroscopic characterization of **3**–**9** revealed a novel structural motif consisting of a dianionic dihydroanthracene-like tricyclic ring system with a central diazadicarbadizinca (ZnCN)_2_ ring, face-capped on either side by PMDETA-wrapped K^+^ cations. All the new metalated pyridine complexes share this dimeric arrangement. As determined by NMR spectroscopic investigations of the reaction filtrates, those solutions producing **3**, **7**, **8**, and **9** appear to be essentially clean reactions, in contrast to those producing **4**, **5**, and **6**, which also contain laterally zincated coproducts. In all of these metalation reactions, the potassium–zincate base acts as an amido transfer agent with a subsequent ligand-exchange mechanism (amido replacing alkyl) inhibited by the coordinative saturation, and thus, low Lewis acidity of the 4-coordinate Zn centers in these dimeric molecules. Studies on analogous trialkyl–zincate reagents in the absence and presence of stoichiometric or substoichiometric amounts of TMP(H) established the importance of Zn–N bonds for efficient zincation.

## Introduction

Although they belong to one of the oldest known organometallic families, alkali–zincate compounds of mixed alkylamido formulation are among the youngest established potent organometallic bases. Many aromatic and heteroaromatic substrates, which are generally inert to familiar neutral organozinc compounds (R_2_Zn) can now be selectively metalated with this new, improved generation of zincate reagent [M^+^(R_2_NZnR_2_)^−^].[1] Often superior in terms of functional-group tolerance, compatibility with more organic substrates, and milder experimental conditions than classical lithiation methods, these new metalations, which are zinc–hydrogen exchange reactions assisted by the presence of a charge-balancing alkali metal cation, can be interpreted as “alkali-metal-mediated zincations” (AMMZn).[2] A range of other mildly electropositive, soft metals including aluminum,[3] cadmium,[4] iron,[5] magnesium,[6,1] and manganese[7] have similarly been transformed into powerful selective metalating agents through this special alkali-metal-mediated phenomenon. Two widely studied reagents in the context of AMMZn are TMP based, one being the lithium–zincate [Li(tmp)Zn(*t*Bu)_2_] (TMP=2,2,6,6-tetramethylpiperidide) developed by Kondo and Uchiyama[8,^2c^] and the other is the sodium analogue [(tmeda)Na(μ-*t*Bu)(μ-tmp)Zn(*t*Bu)] (TMEDA=*N*,*N*,*N′*,*N′*-tetramethylethylenediamine) made by our own group,^[2b]^ whereas Knochel has recently introduced an efficient zincating reagent by mixing the lithium–magnesiate Mg(tmp)_2_**⋅**2LiCl with the halide ZnCl_2_.[9]

Although synthetically, structurally, and mechanistically the use of these lithium– and sodium–TMP–zincate reagents in AMMZn applications is largely well understood, little comparable information is available on potassium–TMP–zincate chemistry. Because the alkali metal plays a pivotal, albeit supporting, role in AMMZn methodology, and given that major distinctions exist between certain lithium, sodium, and potassium congeners in other classes of organometallic compounds, we decided to develop a complementary TMP–zincate chemistry of potassium. Our previously reported starting point was to introduce the diethyl derivative of the potassium–TMP–zincate [(pmdeta)K(μ-Et)(μ-tmp)Zn(Et)] (**1**) (PMDETA=*N*,*N*,*N′*,*N′′*,*N′′*-pentamethyldiethylenetriamine) and to establish its potential as a synergic (potassium-zinc cooperative) zincating base through preliminary reactions with 4-(dimethylamino)pyridine and 4-methoxypyridine.[10] Herein, in an extension and elaboration of previous work, we report a systematic study of **1** with a wide range of pyridine substrates, comparing and contrasting its behavior in both stoichiometric and catalytic reactions with those of related potassium–dialkyl–TMP–zincates and potassium–trialkyl–zincates (TMP free). Significantly, this study uncovers important fundamental differences between **1** and analogous lithium– and sodium–TMP–zincates, underlining the fact that alkali-metal effects that are predominately structural in origin must be explicitly taken into account to attain a full explanation of zincate chemistry.

## Results and Discussion

**Scheme 1:**
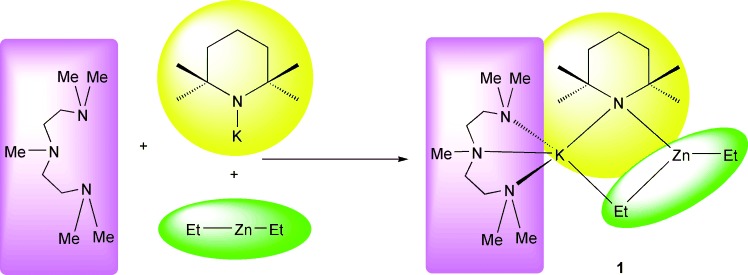
Interlocking co-complexation synthesis of the new synergic base 1.

**Synthesis of the new potassium–zincate bases**: The same reaction methodology was employed to prepare each of the desired potassium–TMP–zincate bases [(pmdeta)K(μ-R)(μ-tmp)Zn(R)] (**1**, R=Et; **2**, R=*n*Bu) (Scheme 1). Trimethylsilylmethylpotassium, Me_3_SiCH_2_K, proved to be a convenient potassium source. Previously, it has been made by reduction of bis(trimethylsilylmethyl)mercury with potassium metal under subambient conditions, but this method affords a hazardous mercury amalgam byproduct.[11] Our safer ambienttemperature approach was a metathetical precipitation reaction between the lithium congener Me_3_SiCH_2_Li and the alkoxide *t*BuOK, from which Me_3_SiCH_2_K was obtained as a white solid in a good isolated yield of 93%. Its purity was established by ^1^H NMR spectroscopy. Next, Me_3_SiCH_2_K was converted to (pmdeta)K(tmp) by initial addition of the amine TMP(H) followed by PMDETA (or vice versa), and the interlocking co-complexation process (Scheme 1) was completed by addition of the appropriate dialkylzinc (Et_2_Zn or *n*Bu_2_Zn) reagent. Diethyl reagent **1** and the butyl homologue **2** were obtained in solid (colorless and crystalline) form (in isolated yields of 61 and 20%, respectively) and were also identified from easily assignable ^1^H and ^13^C NMR spectra (see the Experimental Section for details). Adding to the database of potassium–zincate structures,[12] which is relatively sparse compared with those of other categories of alkali-metal organometallic compounds, the previously reported molecular structure of **1**[10] is depicted in Scheme 1. Designed based upon a KNZnC ring of four different atoms, terminal PMDETA (3×N) and Et (1×C) ligands on K and Zn, respectively, complete the structure. Substituting Et ligands by *n*Bu may be dimensionally significant, but the gross molecular structure of **2** is essentially equivalent to that of **1**.

**Scheme 2:**
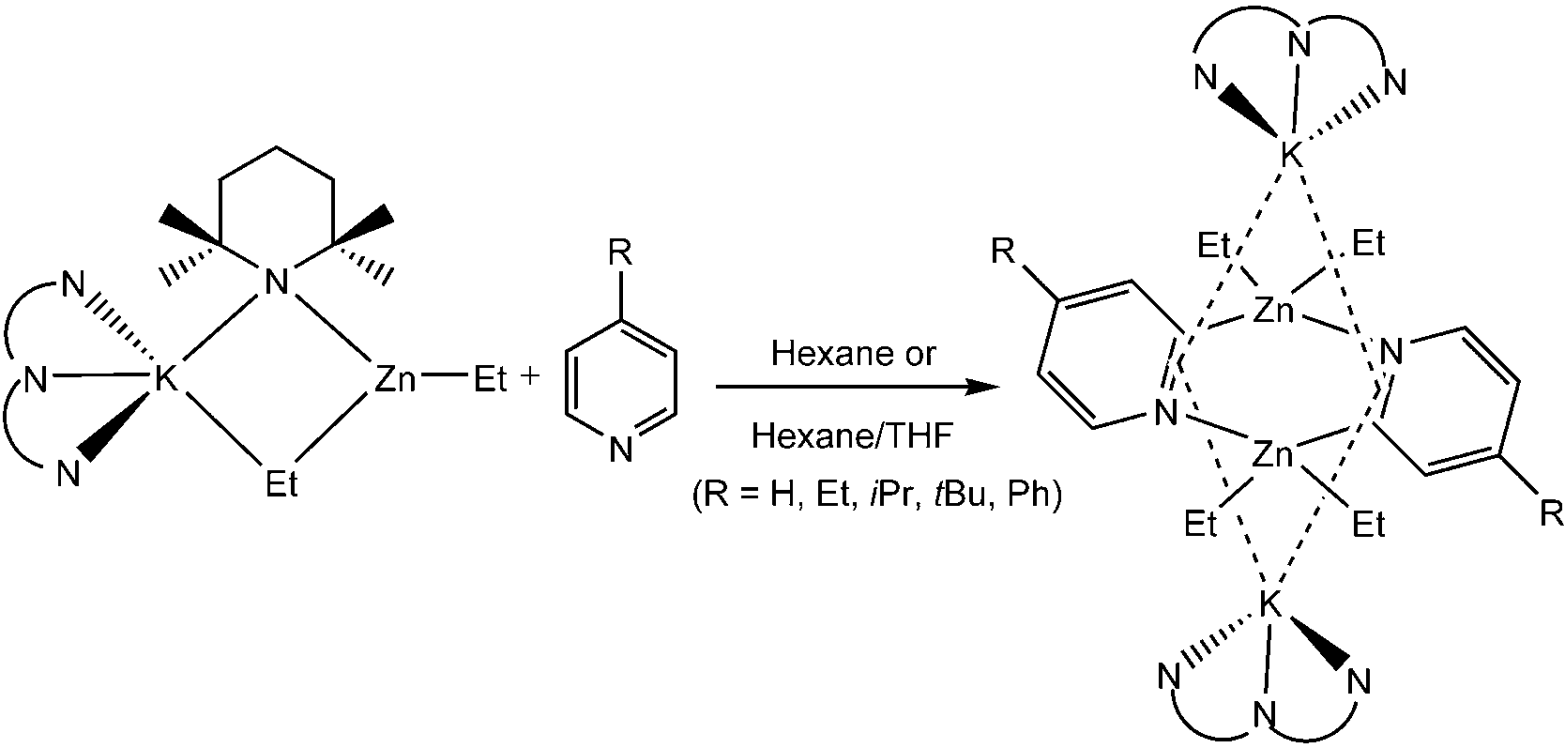
Zincation reactions of 4-substituted pyridines.

**AMMZn reactions of pyridine substrates**: Our initial objective was to accumulate definitive structural information on the zincated intermediates formed from this series of reactions, so the focus was on growing, from solution, suitable crystals for single-crystal X-ray crystallographic characterization. Note, however, that the reaction filtrates obtained following isolation of the crystalline products have also been probed by NMR spectroscopic studies (see later). Following the general protocol shown in Scheme 1, the base **1**, prepared in situ in hexane solution, was treated with one molar equivalent of 4-dimethylaminopyridine, pyridine, 4-ethylpyridine, 4-isopropylpyridine, 4-*tert-*butylpyridine, or 4-phenylpyridine. These reactions yielded the crystalline products [{2-Zn(Et)_2_-μ-4-R-C_5_H_3_N}_2_**⋅**2{K(pmdeta)}] (R=Me_2_N (**3**), H (**4**), Et (**5**), *i*Pr (**6**), *t*Bu (**7**), and Ph (**8**)) in isolated yields of 53, 16, 7, 23, 67, and 51%, respectively (Scheme 2). In addition, reaction of the dibutyl reagent **2** with 4-*tert*-butylpyridine afforded a 58% crystalline yield of [{2-Zn(*n*Bu)_2_-μ-4-*t*Bu-C_5_H_3_N}_2_**⋅**2{K(pmdeta)}] (**9**). Owing to the close similarity between **9** and the family of diethyl analogues, no attempts were made to grow more crystalline complexes from reactions of **2** with other pyridine substrates because it was assumed that **9** would be representative of a common structural motif. All of these new potassium–zincates were characterized by single-crystal X-ray crystallography, and solutions of the potassium–zincates, as well as reaction filtrates (following removal of the crystalline products), were analyzed by ^1^H and ^13^C NMR spectroscopy. These results are now discussed in turn.

**Figure 1:**
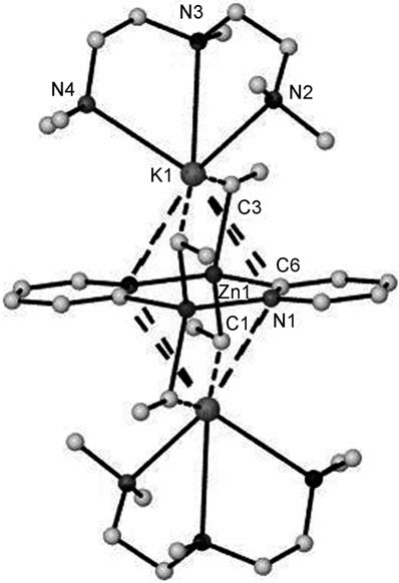
Molecular structure of the 2-zincated pyridine compound [{2-Zn(Et)_2_-μ-4-H-C_5_H_3_N}_2_⋅2{K(pmdeta)}] 4. Minor disorder and hydrogen atoms are omitted for clarity. The dashed line represents potassium–η^2^-N,C interactions with the pyridine rings.

**Figure 2:**
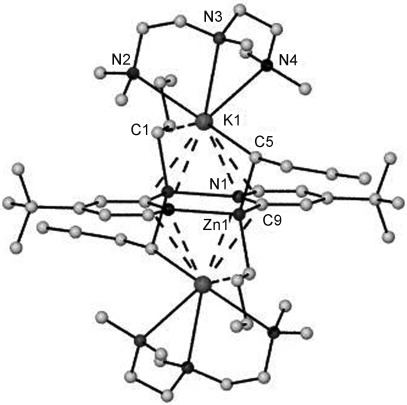
Molecular structure of the 2-zincated pyridine compound [{2-Zn(*n*Bu)_2_-μ-4-*t*Bu-C_5_H_3_N}_2_⋅2{K(pmdeta)}] 9. Minor disorder and hydrogen atoms are omitted for clarity.

**Solid-state structures**: The diethyl–zincate family and their dibutyl analogue **9** share a common dimeric structural motif. For brevity, only the specific structures of **4** and **9** are shown (Figures 1 and 2, respectively) because they are representative of the complete series **3**–**9**. As in the case of previously reported **3**,[10] structures **4**–**9** can be viewed as heterocyclic 9,10-dihydroanthracene mimics, each with a central diazadicarbadizinca (ZnCN)_2_ ring, capped above and below by a potassium cation, which is complexed with a PMDETA ligand. All of these discrete molecules sit on crystallographically imposed inversion centers. Note that there are two, nearly identical, independent molecules of **8** in the asymmetric unit of the crystal structure. Across the series, the central hexagonal (ZnCN)_2_ ring is essentially planar and lies in the same plane as the two outer pyridyl rings as illustrated by the small dihedral angles between the pyridyl ring plane and the central (ZnCN)_2_ ring plane (the range for **3**–**7** and **9** is 1.0(8)–2.93(15) and 1.2(4)°, respectively), although the values for **8** are slightly larger (5.80(19) and 12.52(14)°, respectively). In **8** the phenyl rings are also twisted out of the pyridine ring plane (torsion angles for C26-C27-C30-C31 and C2-C3-C6-C7 are 39⋅0(5) and 34.4(5)°, respectively. See the Supporting Information for the atom labeling scheme). Within each structure, the monomeric unit of the anthracene-like dianionic system comprises a pyridine molecule substituted at the 2-position by a diethylzinc molecule (indicative of the zinc–hydrogen exchange process inherent in AMMZn), with the dimerization junction being comprised of antiparallel (head-to-tail) dative Zn–N(pyr) bonds (pyr=pyridine). Overall, zinc occupies a modestly distorted tetrahedral C_3_N environment (with a range of the mean bond angles across the series from 109.22 to 109.34°). Nine atoms (C×4 and N×5) fill the coordination sphere of the larger potassium cation comprising two nonequivalent η^2^-N,C interactions with the pyridyl rings (involving the anionic deprotonated C center), which leads to an unsymmetrically bound tridentate PMDETA ligand, and contacts to two α-C atoms of two Et or *n*Bu ligands.

Remarkably little variation is observed in the comparative dimensions of structures **3**–**8**. The mean lengths of the “anionic” Zn–C bonds cover the narrow range 2.067–2.1007 Å (Δ=0⋅0337 Å), all of which are shorter than the Zn–N dative bonds (in the range 2.167(4)–2.280(16) Å; Δ 0.061 Å). In line with increasing steric demands, the shortest Zn–N bond involves substituent-free pyridine, whereas the longest Zn–N bond involves the 4-*t*Bu-substituted derivative. Interactions between the potassium ion and the central (ZnCN)_2_ ring are generally biased towards one N,C unit and form shorter contacts (e.g., in **3**, K–N, 3.036(13) Å; K–C, 3.1139(16) Å) than with the other (K–N, 3.2905(13) Å; K–C, 3.2791(15) Å). Potassium also engages in long, weak, electron-deficient interactions with the zinc-bound ethyl ligands (in the range of K–C_α_ lengths 3.140(2)–3.3896(17) Å). Significantly shorter are the dative K–N (PMDETA) bonds, which cover the range 2.8422(14)–3.0512(19) Å.

The molecular architecture shared by structures **3**–**9** appears to be unique compared with any metalated pyridine derivative. There are a handful of literature compounds that possess a similar dihydroanthracene-type polycyclic arrangement. For example, p-block dimethylaluminum and -gallium complexes of unsubstituted pyridine, [Me_2_E(μ-pyr)]_2_ (E=Al or Ga), have central (NCE)_2_ shallow boat rings.[13] The d-block complex [(Br)(PPh_3_)Pd(μ-pyr)]_2_[14] and the s-block complex [(Br)(thf)Mg(μ-pyr)]_2_.(μ-thf)][15] also share this dimeric tricyclic motif. For specific comparison with **3**, metalated 4-dimethylaminopyridine structures are limited to two neutral Al complexes exhibiting a (NCAl)_2_ flattened chair conformation.[16] However, none of these pyridyl dimers are heterobimetallic or ate complexes, nor do they show any type of face-capping similar to that observed here with potassium, because their (NCE)_2_ faces lie vacant. Moreover, structurally defined 2-zincated pyridines are especially rare. [{Zn[Si(NMe_2_)_2_(NHCMe_3_)(NCMe_3_)](μ-NC_5_H_4_)}_2_] is the one previous example, which was made by an indirect metathetical approach and not by direct zincation, and also displays an uncapped (NCE)_2_ (E=Zn) tricyclic arrangement.[17] Without the extra coordination provided by potassium caps, the (mean) Zn–C and Zn–N(pyr) bonds are predictably shorter (by 0.163 and 0.089 Å, respectively) in this neutral zinc complex compared with that in the ate example **4**. To the best of our knowledge, there are no previous crystal structures for zincated, or indeed any metalated, 4-ethyl-, 4-isopropyl- or 4-phenylpyridines, thus structures **5**, **6**, and **8** represent the first of their type. This previous dearth of crystallographic structural information markedly contrasts with the large body of studies in which metalated pyridines have been quenched (for example, electrophilically) and thus studied indirectly without isolation.[18] Two advantages of AMMZn are clearly evident from this comparison: first, that the method often facilitates the formation of crystalline intermediates, and second, that the higher stability of zincated pyridines in relation to more polar metalated pyridines (typically lithopyridines) makes them more amenable to isolation from solution under mild conditions without decomposing, which enables their crystallographic characterization. At the very least, these crystal structures represent resting states of possible solution structures.

**Solution studies**: Potassium–zincates **3**–**9** are highly soluble in arene solvents, enabling the recording of their ^1^H and ^13^C NMR spectra from C_6_D_6_ solution. In all cases only one set of resonances, consistent with the crystallographically determined formulae, were observed. Table 1 compares the ^1^H NMR chemical shifts of the aliphatic resonances of the diethyl–zincates **3**–**8** with standards of the base **1**, diethylzinc, and PMDETA. Without exception, the CH_2_ and CH_3_ (Et) resonances of the potassium–zincates shift upfield and downfield, respectively, with respect to those of neutral diethylzinc. Bimetallic base **1** retains much of its parent zinc character because its CH_2_ (Et) resonance (*δ*=0.47 ppm) lies close to that of diethylzinc (*δ*=0.55 ppm), whereas those of **3**–**8** lie more upfield in the narrow range *δ*=0.15–0.24 ppm. More remote from the zinc center, the CH_3_ (Et) resonances of **1** and **3**–**8** are grouped together in the range *δ*=2.03–2.15 ppm. PMDETA resonances for **1** and **3**–**8** shift upfield (in the range *δ*=1.71–2.01 ppm) compared with those of the free ligand (*δ*=2.11–2.46 ppm), which is indicative of the PMDETA–K^+^ chelation observed in the crystal structure. Consistent with a pyridine zincated in the 2-position, only four aromatic resonances at *δ*=8.93, 8.05, 7.03, and 6.66 ppm are found in the ^1^H NMR spectrum of **4**. The ^13^C NMR spectra concur with this, with the 2-zincated C resonance appearing downfield at *δ*=150.55 ppm. In general, there is little discrimination between the common resonances in the ^13^C NMR spectra of **3**–**8** with, for example, the CH_2_ (Et) resonance covering the small range (*δ*=3.87 in **5** to 6.12 ppm in **8**). These are substantially shifted downfield compared with that in the standard diethylzinc (*δ*=−2.12 ppm), which reflects both aggregative dimer/monomer and zinc coordination-sphere (C_3_N versus C_2_) differences. Displaying a unique trigonal planar (C_2_N) zinc geometry within the series, **1** exhibits the most downfield CH_2_ (Et) signal of all at *δ*=8.99 ppm. The complete assignment of ^1^H and ^13^C NMR spectra is listed in the Experimental Section.

**Table 1:** Selected ^1^H NMR chemical shifts of the starting materials, the zincate base 1, and zincated pyridine products 3–8 in solutions of C_6_D_6_.

Compound	^1^H NMR (CH_2_, Et) [ppm]	^1^H NMR (CH_3_, Et) [ppm]	^1^H NMR (4×CH_3_, PMDETA) [ppm]	^1^H NMR (1×CH_3_, PMDETA) [ppm]	^1^H NMR (4×CH_2_, PMDETA) [ppm]
Et_2_Zn	0.55	1.51	–	–	–
PMDETA	–	–	2.11	2.18	2.46, 2.35
**1**	0.47	2.03	1.81	1.74	1.77
**3**	0.22	2.13	1.88	2.01	1.96
**4**	0.15	2.05	1.77	1.95	1.90
**5**	0.19	2.05	1.83	1.97	1.89
**6**	0.17	2.08	1.78	1.98	1.92
**7**	0.15	2.07	1.71	1.96	1.88
**8**	0.24	2.15	1.71	1.94	1.88–1.81

**Figure 3:**
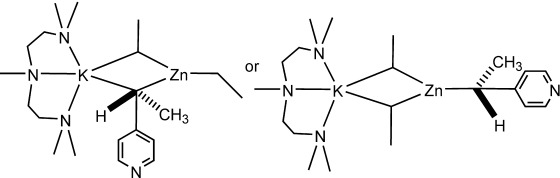
Possible structures of the product of the lateral metalation of 4-ethylpyridine.

Some of the modest-to-poor yields of the new crystalline potassium–zincates (for example, 16 and 7% for **4** and **5**, respectively) suggested that the AMMZn reactions may not be clean, so the reaction filtrates that remained after isolating the crystalline products were also probed by NMR spectroscopy. In the case of **4**, the ^1^H NMR spectra indicated that the oily filtrate appeared to contain a complicated mixture of products. The aromatic region revealed several overlapping resonances in the range *δ*=9.0–6.6 ppm, which proved to be indecipherable. Complex **5** was found to be a minor product, because the major component of the oily, viscous filtrate contained a laterally metalated pyridine molecule. In **5**, the CH_2_ of the Et pyridine substituent had been metalated to CH^−^, with the resonance appearing significantly downfield (*δ*=3.98 ppm) compared with the CH_2_ resonance in 4-ethylpyridine (*δ*=2.20 ppm). This movement of the CH resonance favorably corresponds to those seen in the ^1^H NMR spectra of the isolated lithiated intermediates of the reactions between 4-dimethylaminomethylpyridine and 4-trimethylsilylmethylpyridine with lithium diisopropylamide in THF or Et_2_O.[19] It is likely, given that this lateral metalation of 4-ethylpyridine has metal-bound PMDETA (4×CH_3_ at *δ*=2.03, 1×CH_3_ at 2.00, and 4×CH_2_ at 1.96 ppm) and Et (CH_2_ at *δ*=0.30 and CH_3_ at 1.85 ppm) resonances associated with it, that this major product could be formulated as [(pmdeta)K(μ-4-CH_3_CHC_5_H_4_N)(μ-Et)Zn(Et)] or [(pmdeta)K(μ-Et)_2_Zn(4-CH_3_CH-C_5_H_4_N)] (**10**) and would adopt the same template design as **1** (Figure 3). This result is not surprising because there has been a previous report of the metalation of the side chain of 4-ethylpyridine by using the sodium reagent NaNH_2_ in liquid ammonia.[20] On the basis of similar NMR spectroscopic evidence, the reaction of 4-isopropylpyridine with **1** follows a similar course, with the minor product being crystalline **6** (23% yield) and the major product (found in the oily filtrate) being a laterally metalated pyridine compound, presumably [(pmdeta)K(Et)_2_Zn(4-Me_2_C-C_5_H_4_N)] (**11**). There have also been precedents for CH(*i*Pr) metalation of 4-isopropylpyridine with lithium, sodium, or potassium metal, the intermediates of which were used to carry out side-chain alkenylation and aralkylation reactions with conjugated dienes or styrenes.[21] Lateral metalation of 4-*tert*-butylpyridine is a much more challenging task, so unsurprisingly **7** was the only metalated product in crystalline form (67% yield) or in the reaction filtrate. The treatment of **1** with 4-phenylpyridine also appeared to be clean to give a crystalline yield of **8** of 51%, with more **8** present in the reaction filtrate. Considering this result was achieved by using a base/pyridine stoichiometry of 1:1, it favorably compares with report by Gros and Fort of the lithiation of the same pyridine by using Caubére’s base (“BuLiLiDMAE”), which gives excellent yields (>80%) of the 2-substituted pyridine following electrophilic quenching, but only upon addition of four equivalents of the base.[22] Note that no metalated intermediates were characterized in this earlier study.

Although AMMZn has been accomplished with all of the substrates studied and valuable structural information has been gathered, selectivity is an issue. Ring zincation competes with lateral zincation (in the cases of pyridine R=H, Et, and *i*Pr) and base **1** is not satisfactory because it gives a mixture of products. On the other hand, when R=Me_2_N, *t*Bu, and Ph, base **1** is a highly effective zincator, achieving 2-zincation selectively under mild (ambient temperature) conditions.

**Scheme 3:**
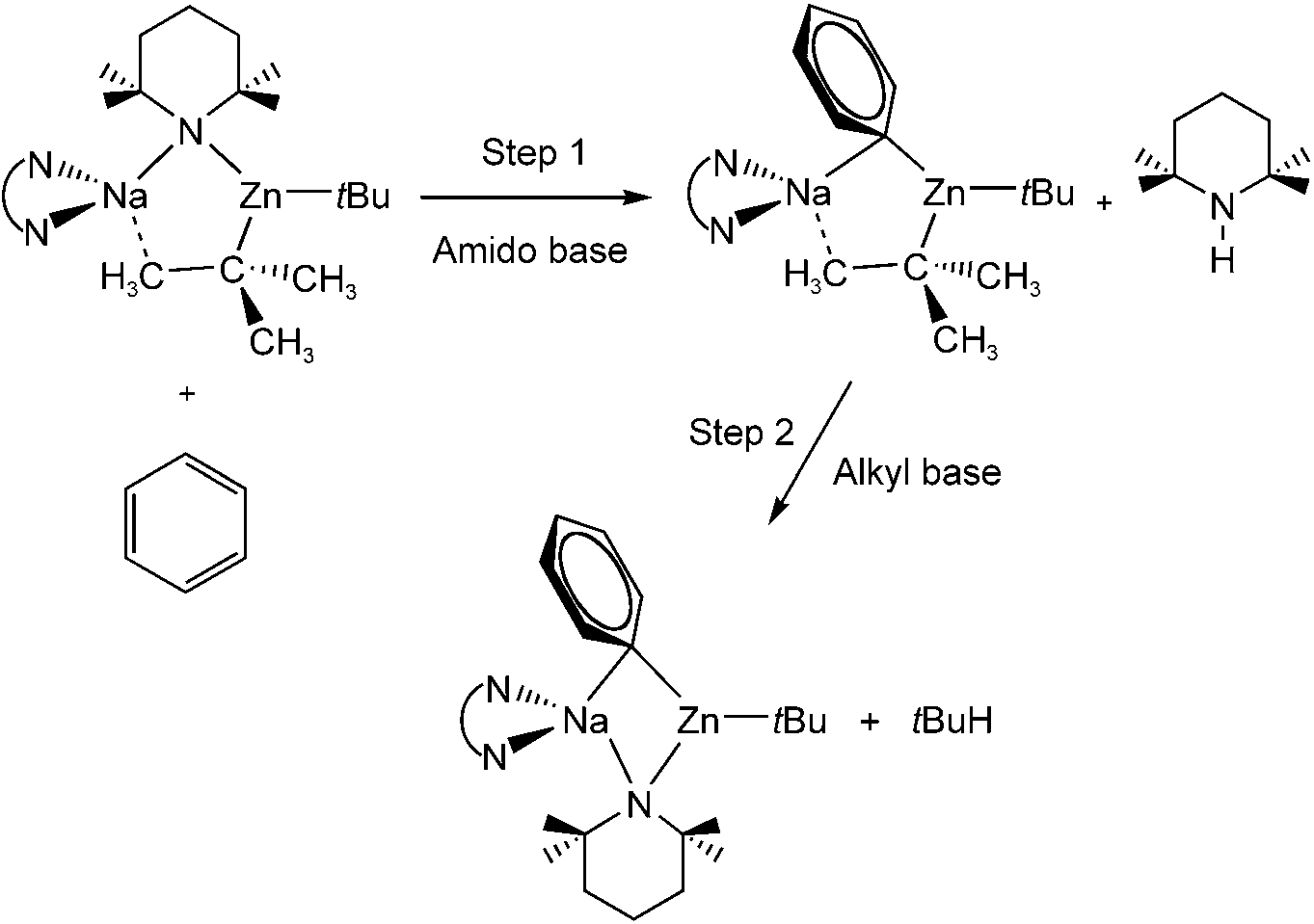
Proposed two-step reaction for AMMZn of benzene.

**Ligand transfer and catalytic considerations**: Because it possesses a heteroleptic formulation, **1** could, in theory, behave as an amido (TMP) or alkyl (Et) base (or both). This is one of the engrossing features of this class of alkylamido reagent. All of the evidence accumulated from this study implies that **1** is exclusively a TMP base. Thus, no TMP ligands are found in the structures of **3**–**9**, but TMP(H) is observed in the reaction filtrates. This appears to contrast with AMMZn reactions in which AM=Li or Na, which generally, although not exclusively, ultimately act as alkyl bases. By exploiting key structures previously elucidated by X-ray crystallography to build models for theoretical investigation, Uchiyama et al. showed through DFT calculations that lithium– or sodium–TMP–dialkyl–zincate reagents deprotonate substrates in two steps: in step 1, TMP abstracts a proton from the substrate to form TMP(H); in step 2, TMP(H) is deprotonated to TMP, which forms part of the deprotonated substrate complex, and alkane is concomitantly released (Scheme 3).

**Scheme 4:**
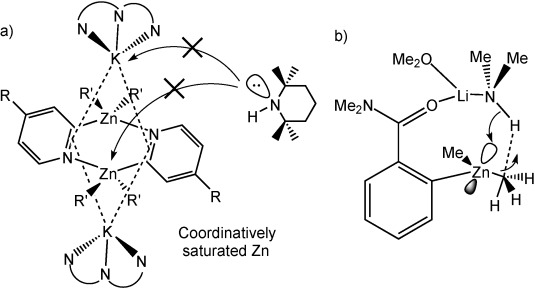
Contrasting coordination-number dependent reactivity of zincates with amines.

Admittedly, this is not a direct comparison with the pyridine substrate investigations because the theoretical approach considered only anisole,^[8a]^ benzene,[23] benzonitrile,[24] methyl benzoate,[24] and *N*,*N*-diisopropylbenzamide substrates.[25] Further to these theoretical studies of Uchiyama et al., we obtained direct experimental evidence of this two-step mechanism, revealing that step 1 can also go backwards to regenerate the starting base and substrate, and that the course of these reactions critically depends on the identity of the alkyl ligand (e.g., Me versus *t*Bu) and solvent.[26] It appears that the potassium–zincate reactions reported here stop at step 1, and there could be several different contributing factors behind this such as the higher carbophilicity and softer character of potassium versus lithium or sodium[27] combined with the high covalent character of zinc, which could stabilize the KZnR_2_(pyr) intermediates and preclude any subsequent reaction with TMP(H). However, the main factor is probably structural in origin, because in contrast with the lithium– and sodium–zincate systems, which are dinuclear and monomeric, the potassium–zincates **3**–**9** are tetranuclear and dimeric. Assuming that aggregation occurs faster than TMP(H) “re-coordination”, a PMDETA-wrapped, coordinatively saturated K^+^ center does not have a coordination site available for TMP(H) to rejoin the bimetallic complex, and consequently, no alkyl–alkane/TMP(H)–TMP reaction can take place. Moreover, in contrast with the lithium– and sodium–zincate systems in which zinc exhibits a relatively exposed trigonal planar, 3-coordinate environment, in compounds **3**–**9** the zinc exhibits a tetrahedral, 4-coordinate environment made up of three Zn–C bonds and one Zn–N bond. Therefore, the zinc center in **3**–**9** is coordinatively saturated, and as a result, is of greatly diminished Lewis acidity compared with the coordinatively unsaturated zinc centers in the lithium– and sodium–zincates (Scheme 4). This distinction may not necessarily be an alkali metal (K versus Li or Na) effect because it depends on the structures of the deprotonated substrate complexes formed, which are determined by a combination of factors including the nature of the alkyl ligand, the nature of the amide ligand, the nature of the Lewis-base supporting ligand, as well as the identity of the substrate undergoing deprotonation. Hence, it is imperative to determine the structure of the metal-containing deprotonated substrate complex to gain a full explanation for the ligand-transfer chemistry taking place. Uchiyama et al. recently reported that the TMP–aluminate *i*Bu_3_Al(tmp)Li behaves fundamentally differently from TMP–zincates with respect to the *ortho* metalation of aromatic substrates, in which *i*Bu_3_Al(tmp)Li acts as a TMP base in a single step compared with the two-step ultimate alkyl basicity of the TMP–zincates.[28] Similarly, the idea of diminished Lewis acidity at coordinately saturated, 4-coordinate Al centers, which was supported by DFT calculations, was used to explain the distinction mentioned above. Hence, the Zn centers in **3**–**9** can be viewed as pseudo Al centers, and it can be stated that the mechanism followed by TMP–metalates (1 or 2 step) is not due to any inherent difference between Al and Zn, but is dictated by the structure of the metal-containing deprotonated substrate complex formed in step 1.

The fact that the participation of TMP (and the implication of Zn–N bonds) is essential for the effective formation of AMMZn was confirmed by qualitative experiments by employing 4-*tert*-butylpyridine as a reference substrate. Thus, bases **1** and **2** were found to 2-zincate the pyridine standard (1:1 molar equivalents in hexane solution) almost quantitatively in approximately one hour and a few minutes, respectively, as determined by NMR spectroscopic analysis of reaction aliquots at various time intervals. Upon substituting TMP by the alkyl Me_3_SiCH_2_ to generate in situ [(pmdeta)K(Et)(CH_2_SiMe_3_)Zn(Et)] and [(pmdeta)K(*n*Bu)(CH_2_SiMe_3_)Zn(*n*Bu)] formulations devoid of any Zn–N bonding, the degree of metalation was low, even after a week,[29] amounting to only about 4 and 16% for [(pmdeta)K(Et)(CH_2_SiMe_3_)Zn(Et)] and [(pmdeta)K(*n*Bu)(CH_2_SiMe_3_)Zn(*n*Bu)], respectively. Upon addition of 10 mol% of TMP(H) to these trialkyl–zincate formulations, the metalation levels significantly increased to about 49 and 79%, respectively, although the reactions were still extremely slow with these conversions obtained after approximately 8 and 4 days, respectively. These results suggest that TMP(H) reacts very slowly with the trialkyl–zincates in a rate-determining step, because the products **1** and **2** react comparatively quickly with the pyridine substrate. Because these metalation levels greatly exceed 10%, TMP(H) must be acting catalytically. Zn–N bonds clearly hold the key to these enhanced metalating rates. The superior reactivity of Zn–N bonds versus Zn–C bonds has also been recently documented by Hagadorn et al., who revealed that simple secondary amines (e.g., morpholine, pyrrolidine) in stoichiometric or catalytic quantities greatly increased the rate of Zn–H exchange between Ph_2_Zn and a range of relatively non-acidic carbon substrates (e.g., *N*,*N*-diethylacetamide and trimethylphosphane oxide).[30] The proposed reactive intermediates are neutral arylamido zinc PhZn(NR_2_) formulations, although none were structurally defined in the study. Hagadorn notes that sterically demanding amines, such as *i*Pr_2_N(H) and (Me_3_Si)_2_NH, are ineffective promoters of Zn– H exchange because they do not form zinc amides with Ph_2_Zn at a reasonable rate; a limitation comparable with the slow TMP(H)–trialkyl–zincate reactions observed here. When the trialkyl–zincates were treated with one molar equivalent of TMP(H), similar metalation levels were achieved (49 and 86%, respectively) and, although still slow, the reaction rates to obtain these conversions (about 27 and 19 h, respectively) were much improved in comparison to those obtained by using substoichiometric amounts of TMP(H). A final point to note is that co-complexation of Me_3_SiCH_2_K with Et_2_Zn actually leads to a significant diminishment of the metalating power of the potassium alkyl, because on its own it reacts with TMP(H) almost instantaneously to form KTMP.[31]

## Conclusion

Potassium-mediated zincation has been studied in the context of pyridine metalation chemistry. By utilizing a potassium–dialkyl–TMP–zincate base, zinc has been delivered regioselectively and directly to the 2-position of the heterocyclic ring in substituted 4-R-pyridines, in which R is Me_2_N, *t*Bu, or Ph, but the new methodology is less successful when R is H, Et, or *i*Pr, because mixtures of ring- and laterally zincated products are produced. Seven of the zincated pyridine intermediate (that is, existing before any electrophilic-quenching protocol) complexes have been structurally defined by single-crystal X-ray crystallography and NMR spectroscopy, and have been found to share a common dimeric motif in which a central diazadicarbadizinca (ZnCN)_2_ dianionic ring is capped on either side by a PMDETA-wrapped K^+^ cation. The coordinative saturation of the distorted tetrahedral Zn center within these structures appears to be a major factor in the inhibition of a subsequent alkyl–amine/alkane–amido ligand transfer that is commonly observed in lithium– and sodium–dialkyl–TMP–zincate reactions.

## Experimental Section

**Methods and materials**: All reactions and manipulations were performed by using standard Schlenk techniques under argon gas. Products were isolated inside an argon-filled dry box. Solvents were freshly distilled from sodium/benzophenone prior to use. TMP(H) was obtained from Aldrich and dried over 4 Å molecular sieves before use. Other amines were obtained from Aldrich, distilled from CaH_2_ and stored over 4 Å molecular sieves. All other chemicals were obtained from Aldrich and used as supplied. ^1^H and ^13^C NMR spectra were recorded on a Bruker DPX 400 MHz spectrometer. All ^13^C NMR spectra were proton decoupled. Correlations between protons and carbon atoms were obtained through COSY and HSQC NMR spectroscopic methods. Single-crystal X-ray diffraction data were recorded on Nonius Kappa CCD and Oxford Diffraction Gemini A Ultra diffractometers using graphite-monochromated Mo_Kα_ radiation (0.71073 Å) (see the Supporting Information for a table of selected crystallographic data).[32] The structures were solved by direct methods (SHELX-97 or SIR program package) and refined on all unique *F*^2^ values (SHELX).[33] CCDC-683512 (**1**), 721902 (**2**), 683513 (**3**), 721903 (**4**), 721904 (**5**), 721905 (**6**), 721906 (**7**), 721907 (**8**), and 721908 (**9**) contain the supplementary crystallographic data for this paper. These data can be obtained free of charge from The Cambridge Crystallographic Data Centre via http://www.ccdc.cam.ac.uk/data_request/cif.

**Synthesis of KCH_2_Si(CH_3_)_3_**: KO*t*Bu (2.75 g, 25 mmol) was dissolved in hexane (50 mL) in a Schlenk tube. A solution of LiCH_2_Si(CH_3_)_3_ (1m; 25 mL, 25 mmol) in pentane was added, and the reaction mixture was left to stir overnight to form an off-white suspension. The solid was filtered, washed with hexane (2×20 mL), and dried in vacuo to afford a white solid (2.80 g, 93% yield). ^1^H NMR (400.13 MHz, 298 K, [D_8_]THF): *δ*=−0.20 (s, 9H; 3×CH_3_), −2.24 ppm (s, 2H; CH_2_K).

**Synthesis of [(pmdeta)K(μ-Et)(μ-tmp)Zn(Et)] (1)**: KCH_2_Si(CH_3_)_3_ (0.24 g, 2 mmol) was suspended in hexane (10 mL). PMDETA (0.84 mL, 4 mmol) was added to afford a clear orange solution before the addition of TMP(H) (0.34 mL, 2 mmol) and a solution of Et_2_Zn (1m; 2 mL, 2 mmol) in hexane. The Schlenk tube was placed in a freezer (−28 °C) overnight to afford colorless crystals (0.58 g, 61% yield). ^1^H NMR (400.13 MHz, 298 K, C_6_D_6_): *δ*=2.06–1.98 (m, 8H; 2×CH_3_ Et and γH TMP), 1.81 (s, 12H; 4×CH_3_ PMDETA), 1.77–1.73 (m, 11H; 4×CH_2_ and 1×CH_3_ PMDETA), 1.58 (t, *J*=6.0 Hz, 4H; H_β_ TMP), 1.36 (s, 12H; 4×CH_3_ TMP), 0.47 ppm (q, *J*=8.0 Hz, 4H; 2×CH_2_ Et); ^13^C{^1^H} NMR (100.62 MHz, 298 K, C_6_D_6_): *δ*=56.9 (2×CH_2_ PMDETA), 55.2 (2×CH_2_ PMDETA), 45.2 (4×CH_3_ PMDETA), 41.6 (1×CH_3_ PMDETA), 41.2 (2×C_β_ TMP), 35.1 (4×CH_3_ TMP), 20.8 (1×C_γ_ TMP), 15.1 (2×CH_3_ Et) 9.0 ppm (2×CH_2_ Et).

**Synthesis of [(pmdeta)K(μ-*n*Bu)(μ-tmp)Zn(*n*Bu)] (2)**: KCH_2_Si(CH_3_)_3_ (0.24 g, 2 mmol) was suspended in hexane (10 mL). PMDETA (0.42 mL, 2 mmol) was added to afford a clear orange solution to which TMP(H) (0.34 mL, 2 mmol) and a solution of *n*Bu_2_Zn (1m; 2 mL, 2 mmol) in heptane were added. The Schlenk tube was placed in a freezer (−28°C) overnight to afford colorless crystals (0.21 g, 19.8% yield). ^1^H NMR (400.13 MHz, 298 K, C_6_D_6_): *δ*=2.25 (quintet, *J*=7.6 Hz, 4H; 2×CH_2_ *n*Bu), 2.03–1.98 (m, 2H; H_γ_ TMP), 1.87–1.78 (m, 15H; 4×CH_2_ PMDETA, 1×CH_3_ PMDETA, 2×CH_2_ *n*Bu), 1.76 (s, 12H; 4×CH_3_ PMDETA), 1.56 (t, *J*=6.0 Hz, 4H; H_β_ TMP), 1.36 (s, 12H; 4×CH_3_ TMP), 1.27 (t, *J*=7.3 Hz, 6H; 2×CH_3_ *n*Bu), 0.41 ppm (t, *J*=8.0 Hz, 4H; 2×CH_2_–Zn *n*Bu); ^13^C{^1^H} NMR (100.62 MHz, 298 K, C_6_D_6_): *δ*=56.9 (2×CH_2_ PMDETA), 55.1 (2×CH_2_ PMDETA), 45.1 (4×CH_3_ PMDETA), 41.6 (1×CH_3_ PMDETA), 41.2 (2×C_β_ TMP), 35.1 (4×CH_3_ TMP), 34.0 (2×CH_2_ *n*Bu), 31.3 (2×CH_2_ *n*Bu), 20.8 (1×C_γ_ TMP), 19.3 (2×Zn–CH_2_ *n*Bu), 14.9 ppm (2×CH_3_ *n*Bu).

**Synthesis of [{2-Zn(Et)_2_-μ-4-Me_2_N-C_5_H_3_N}_2_⋅2{K(pmdeta)}] (3)**: KCH_2_Si(CH_3_)_3_ (0.24 g, 2 mmol) was suspended in hexane (10 mL). PMDETA (0.84 mL, 4 mmol) was added to afford a clear orange solution to which TMP(H) (0.34 mL, 2 mmol) and a solution of Et_2_Zn (1m; 2 mL, 2 mmol) in hexane were added. 4-(Dimethylamino)pyridine (0.244 g, 2 mmol) was added to form a yellow solution. After 4 h, the solution turned cloudy and THF (2 mL) was added to form a clear solution. The Schlenk tube was placed in a freezer (−28°C) for 3 h to afford colorless crystals (0.48 g, 53% yield). ^1^H NMR (400.13 MHz, 298 K, C_6_D_6_): *δ*=8.74 (brs, 1H; aromatic H), 7.41 (brs, 1H; aromatic H), 6.09 (brs, 1H; aromatic H), 2.55 (s, 6H; 2×N–CH_3_) 2.13 (t, *J*=7.8 Hz, 6H; 2×CH_3_ Et), 2.01 (s, 3H; CH_3_ PMDETA), 1.98–1.94 (m, 8H; 4×CH_2_ PMDETA), 1.88 (s, 12H; 4×CH_3_ PMDETA), 0.22 ppm (q, *J*=7.8 Hz, 4H; 2×CH_2_ Et), (small amount of free DMAP at *δ*=8.29, 6.28 and 2.27 ppm); ^13^C{^1^H} NMR (100.62 MHz, 298 K, C_6_D_6_): *δ*=154.1 (aromatic C), 150.6 (aromatic C), 150.3 (aromatic C at *δ*_H_=8.74 ppm), 119.6 (aromatic C at *δ*_H_*=*7.41 ppm), 103.6 (aromatic C at *δ*_H_=6.09 ppm), 57.5 (2×CH_2_ PMDETA), 56.1 (2×CH_2_ PMDETA), 45.8 (4×CH_3_ PMDETA), 42.3 (1×CH_3_ PMDETA), 38.6 (2×N–CH_3_ DMAP), 16.6 (2×CH_3_ Et), 5.9 ppm (2×CH_2_ Et).

**Synthesis of [{2-Zn(Et)_2_-μ-C_5_H_4_N}_2_⋅2{K(pmdeta)}] (4)**: KCH_2_Si(CH_3_)_3_ (0.24 g, 2 mmol) was suspended in hexane (10 mL). PMDETA (0.84 mL, 4 mmol) was added to afford a transparent orange solution to which TMP(H) (0.34 mL, 2 mmol) and a solution of Et_2_Zn (1m; 2 mL, 2 mmol) in hexane were added. Pyridine (0.16 mL, 2 mmol) was added to form a yellow solution. After 2 h of continuous stirring, an oil and solution bilayer was produced upon leaving the reaction mixture to stand. The solution was transferred to another Schlenk tube by using a cannula, and the oil was discarded. The Schlenk tube containing the solution was placed in a freezer (−28°C) for 4 days, after which time a small crop of colorless crystals had formed (0.13 g, 16% yield). ^1^H NMR (400.13 MHz, 298 K, C_6_D_6_): *δ*=8.93 (brs, 1H; aromatic H), 8.05 (brs, 1H; aromatic H), 7.03 (brs, 1H; aromatic H), 6.66 (brs, 1H; aromatic H), 2.05 (t, *J*=7.9 Hz, 6H; 2×CH_3_ Et), 1.95 (s, 3H; CH_3_ PMDETA), 1.93–1.87 (m, 8H; 4×CH_2_ PMDETA), 1.77 (s, 12H; 4×CH_3_ PMDETA), 0.15 ppm (q, *J*=7.9 Hz, 4H; 2×CH_2_ Et); ^13^C{^1^H} NMR (100.62 MHz, 298 K, C_6_D_6_): *δ*=150.7 (aromatic C at *δ*_H_=8.93 ppm) 150.6 (metalated C), 136.7 (aromatic C at *δ*_H_=8.05 ppm), 129.8 (aromatic C at *δ*_H_=7.03 ppm), 118.4 (aromatic C at *δ*_H_=6.66 ppm), 57.4 (2×CH_2_ PMDETA), 55.9 (2×CH_2_ PMDETA), 45.2 (4×CH_3_ PMDETA), 42.3 (1×CH_3_ PMDETA), 16.2 (2×CH_3_ Et), 6.0 ppm (2×CH_2_ Et).

**Synthesis of [{2-Zn(Et)_2_-μ-4-Et-C_5_H_3_N}_2_⋅2{K(pmdeta)}] (5)**: KCH_2_Si(CH_3_)_3_ (0.24 g, 2 mmol) was suspended in hexane (10 mL). PMDETA (0.84 mL, 4 mmol) was added to afford a transparent orange solution to which TMP(H) (0.34 mL, 2 mmol) and a solution of Et_2_Zn (1m; 2 mL, 2 mmol) in hexane were added. 4-Ethylpyridine (0.23 mL, 2 mmol) was added to form a yellow solution. After 0.5 h of continuous stirring, an oil and solution bilayer was produced upon leaving the reaction mixture to stand. The solution was transferred to another Schlenk tube through a cannula, and the viscous oil was discarded. The Schlenk tube containing the solution was placed in the freezer (−28°C) overnight to yield a crop of crystals (0.06 g, 7% yield). ^1^H NMR (400.13 MHz, 298 K, C_6_D_6_): *δ*=8.90 (brs, 1H; aromatic H), 7.95 (brs, 1H; aromatic H), 6.58 (brs, 1H; aromatic H), 2.35 (q, *J*=7.3 Hz, 2H; CH_2_ pyr Et), 2.05 (t, *J*=7.9 Hz, 6H; 2×CH_3_ Et), 1.97 (s, 3H; CH_3_ PMDETA), 1.91–1.87 (m, 8H; 4×CH_2_ PMDETA), 1.83 (s, 12H; 4×CH_3_ PMDETA), 1.11 (t, *J*=7.6 Hz, 3H; CH_3_ pyr Et), 0.19 ppm (q, *J*=8.0 Hz, 4H; 2×CH_2_ Et) (some product of lateral metalation as a viscous oil is found at *δ*=7.04, 6.81, 5.70, 5.60, 3.99 and under the multiplet at 1.89 ppm); ^13^C{^1^H} NMR (100.62 MHz, 298 K, C_6_D_6_): *δ*=150.2 (aromatic C at *δ*_H_=8.90 ppm), 136.1 (aromatic C at *δ*_H_=7.95 ppm), 118.0 (aromatic C at *δ*_H_=6.58 ppm), 56.8 (2×CH_2_ PMDETA), 55.6 (2×CH_2_ PMDETA), 44.8 (4×CH_3_ PMDETA), 41.1 (1×CH_3_ PMDETA), 28.5 (1×CH_2_ pyr Et), 15.4 (2×CH_3_ of Et), 14.7 (1×CH_3_ pyr Et), 3.9 ppm (2×CH_2_ Et) (some product of lateral metalation as a viscous oil is found at *δ*=143.9, 141.3, 111.5, 104.0, 80.9 and 12.7 ppm); filtrate: ^1^H NMR (400.13 MHz, 298 K, C_6_D_6_): *δ*=7.04 (d, *J*=5.3 Hz, 1H; aromatic H), 6.82 (d, *J*=5.6 Hz, 1H; aromatic H), 5.66 (d, *J*=6.6 Hz, 1H; aromatic H), 5.57 (d, *J*=5.1 Hz, 1H; aromatic H), 4.02–3.94 (m, 1H; CH met pyr Et), 2.03 (s, 12H; 4×CH_3_ PMDETA), 2.00 (s, 3H; CH_3_ PMDETA), 1.98–1.94 (m, 8H; 4×CH_2_ PMDETA), 1.85 (t, *J*=8.0 Hz, 6H; 2×CH_3_ Et), 1.78 (d, *J*=6.7 Hz, 3H; CH_3_ pyr Et), 0.30 ppm (q, *J*=7.8 Hz, 4H; 2×CH_2_ Et). For comparison, free 4-ethylpyridine gives signals at *δ*=8.47 (d), 6.71 (d), 2.20 (q), and 0.90 ppm (t) by using the same NMR conditions.

**Synthesis of [{2-Zn(Et)_2_-μ-4-*i*Pr-C_5_H_3_N}_2_⋅2{K(pmdeta)}] (6)**: KCH_2_Si(CH_3_)_3_ (0.24 g, 2 mmol) was suspended in hexane (10 mL). PMDETA (0.84 mL, 4 mmol) was added to afford a transparent orange solution to which TMP(H) (0.34 mL, 2 mmol) and a solution of Et_2_Zn (1m; 2 mL, 2 mmol) in hexane were added. 4-Isopropylpyridine (0.26 mL, 2 mmol) was added to form a cloudy suspension. After 2 h of continuous stirring, THF (3 mL) was added, which resulted in a clear solution. The Schlenk tube containing the solution was placed in a freezer (−28°C) yielding a crop of colorless crystals (0.21 g, 23% yield). ^1^H NMR (400.13 MHz, 298 K, C_6_D_6_): *δ*=8.92 (brs, 1H; aromatic H), 7.95 (brs, 1H; aromatic H), 6.57 (brs, 1H; aromatic H), 2.57 (sept, *J*=6.7 Hz, 1H; CH of *i*Pr), 2.08 (t, *J*=7.8 Hz, 6H; 2×CH_3_ Et), 1.98 (s, 3H; CH_3_ PMDETA), 1.94–1.90 (m, 8H; 4×CH_2_ PMDETA), 1.78 (s, 12H; 4×CH_3_ PMDETA), 1.15 (d, *J*=6.9 Hz, 6H; 2×CH_3_ *i*Pr), 0.17 ppm (q, *J*=8.0 Hz, 4H; 2×CH_2_ Et); ^13^C{^1^H} NMR (100.62 MHz, 298 K, C_6_D_6_): *δ*=150.7 (aromatic C at *δ*_H_=8.92 ppm), 150.3 (tertiary or metalated C), 149.6 (tertiary or metalated C), 135.2 (aromatic C at *δ*_H_=7.95), 116.7 (aromatic C at *δ*_H_=6.57), 57.0 (2×CH_2_ PMDETA), 55.5 (2×CH_2_ PMDETA), 45.0 (4×CH_3_ PMDETA), 42.1 (1×CH_3_ of PMDETA), 33.9 (1×CH of *i*Pr), 23.1 (2×CH_3_ of *i*Pr), 16.0 (2×CH_3_ Et), 5.4 ppm (2×CH_2_ Et); filtrate: ^1^H NMR (400.13 MHz, 298 K, C_6_D_6_): important resonances representing lateral metalated 4-isopropylpyridine: *δ*=6.89 (d, *J*=6.4 Hz, 2H; aromatic H), *δ*=5.58 (d, *J*=6.5 Hz, 2H; aromatic H), 1.75 ppm (s, 6H; 2×CH_3_ *i*Pr). For comparison, free 4-isopropylpyridine gives signals at *δ*=8.50 (d), 6.68 (d), 2.42 (sept), and 0.90 ppm (d) by using the same NMR conditions.

**Synthesis of [{2-Zn(Et)_2_-μ-4-*t*Bu-C_5_H_3_N}_2_⋅2{K(pmdeta)}] (7)**: KCH_2_Si(CH_3_)_3_ (0.24 g, 2 mmol) was suspended in hexane (10 mL). PMDETA (0.84 mL, 4 mmol) was added to afford a transparent orange solution. TMP(H) (0.34 mL, 2 mmol) and a solution of Et_2_Zn (1m; 2 mL, 2 mmol) in hexane were added. 4-*tert*-Butylpyridine (0.29 mL, 2 mmol) was added to form a clear solution. After 0.25 h of continuous stirring, the Schlenk tube containing the solution was placed in a freezer (−28°C) yielding a crop of crystals (0.63 g, 67% yield). ^1^H NMR (400.13 MHz, 298 K, C_6_D_6_): *δ*=8.89 (brs, 1H; aromatic H), 8.11 (brs, 1H; aromatic H), 6.70 (brs, 1H; aromatic H), 2.07 (t, *J*=8.0 Hz, 6H; 2×CH_3_ Et), 1.96 (s, CH_3_ 3H; PMDETA), 1.90–1.86 (m, 8H; 4×CH_2_ PMDETA), 1.71 (s, 12H; 4×CH_3_ PMDETA), 1.25 (s, 9H; 3×CH_3_ *t*Bu), 0.15 ppm (q, *J*=8.0 Hz, 4H; 2×CH_2_ Et); ^13^C{^1^H} NMR (100.62 MHz, 298 K, C_6_D_6_): *δ*=151.5 (tertiary or metalated C), 150.4 (aromatic C at *δ*_H_=8.89 ppm), 133.3 (aromatic C at *δ*_H_=8.11), 115.8 (aromatic C at *δ*_H_=6.70 ppm), 57.3 (2×CH_2_ PMDETA), 55.9 (2×CH_2_ PMDETA), 45.2 (4×CH_3_ PMDETA), 42.3 (1×CH_3_ PMDETA), 34.1 (tertiary C of *t*Bu), 30.8 (3×CH_3_ of *t*Bu), 16.4 (2×CH_3_ of Et), 5.7 ppm (2×CH_2_ Et).

**Synthesis of [{2-Zn(Et)_2_-μ-4-Ph-C_5_H_3_N}_2_⋅2{K(pmdeta)}] (8)**: KCH_2_Si(CH_3_)_3_ (0.24 g, 2 mmol) was suspended in hexane (10 mL). PMDETA (0.84 mL, 4 mmol) was added to afford a transparent orange solution to which TMP(H) (0.34 mL, 2 mmol) and a solution of Et_2_Zn (1m; 2 mL, 2 mmol) in hexane were added. 4-Phenylpyridine (0.31 g, 2 mmol) was added to form a clear solution. After 1.5 h of continuous stirring, THF (3 mL) was added to the Schlenk tube, which was then placed in a freezer (−28°C) overnight to yield a crop of crystals (0.50 g, 51% yield). ^1^H NMR (400.13 MHz, 298 K, C_6_D_6_): *δ*=9.13 (brs, 1H; pyr H), 8.46 (brs, 1H; pyr H), 7.67 (d, *J*=7.4 Hz, 2H; 2×phenyl H), 7.23 (t, *J*=7.4 Hz, 2H; 2×phenyl H), 7.15 (d, *J*=7.3 Hz, 1H; phenyl H), 7.02 (brs, 1H; pyr H), 2.15 (t, *J*=8.0 Hz, 6H; 2×CH_3_ Et), 1.94 (s, 3H; CH_3_ PMDETA), 1.88–1.81 (m, 8H; 4×CH_2_ PMDETA), 1.71 (s, 12H, 4×CH_3_ PMDETA), 0.24 ppm (q, *J*=8.0 Hz, 4H; 2×CH_2_ Et); ^13^C{^1^H} NMR (100.62 MHz, 298 K, C_6_D_6_): *δ*=151.2 (aromatic C at *δ*_H_=9.13 ppm), 141.1 (tertiary pyr C or tertiary Ph C), 141.0 (tertiary pyr C or tertiary Ph C), 134.0 (aromatic C at *δ*_H_=8.46 ppm), 129.3 (aromatic C at *δ*_H_=7.23 ppm), 127.9 (aromatic C at *δ*_H_=7.15 ppm), 127.3 (aromatic C at *δ*_H_=7.67 ppm), 116.5 (aromatic C at *δ*_H_=7.02 ppm), 57.30 (2×CH_2_ PMDETA), 56.0 (2×CH_2_ PMDETA), 45.1 (4×CH_3_ PMDETA), 42.1 (1×CH_3_ PMDETA), 16.6 (2×CH_3_ Et), 6.1 ppm (2×CH_2_ Et).

**Synthesis of [{2-Zn(*n*Bu)_2_-μ-4-*t*Bu-C_5_H_3_N}_2_⋅2{K(pmdeta)}] (9)**: KCH_2_Si(CH_3_)_3_ (0.24 g, 2 mmol) was suspended in hexane (10 mL). PMDETA (0.42 mL, 2 mmol) was added to afford a transparent orange solution. TMP(H) (0.34 mL, 2 mmol) and a solution of *n*Bu_2_Zn (1m, 2 mL, 2 mmol) in heptane were added. 4-*tert*-Butylpyridine (0.29 mL, 2 mmol) was added to form a clear solution. After 10 min of continuous stirring, a suspension was formed. THF (2 mL) was added resulting in a clear solution, and the Schlenk tube containing the solution was placed in a freezer (−28°C) to yield a crop of crystals after 4 days (0.61 g, 58% yield). ^1^H NMR (400.13 MHz, 298 K, C_6_D_6_): *δ=*8.89 (brs, 1H; aromatic H), 8.10 (brs, 1H; aromatic H), 6.68 (brs, 1H; aromatic H), 2.31 (quintet, *J*=7.6 Hz, 4H; 2×CH_2_ *n*Bu), 1.95–1.83 (m, 15H; 4×CH_2_ PMDETA, 1×CH_3_ PMDETA, 2×CH_2_ *n*Bu), 1.70 (s, 12H; 4×CH_3_ of PMDETA), 1.33 (t, *J*=7.3 Hz, 6H; 2×CH_3_ of *n*Bu), 1.26 (s, 9H; 3×CH_3_ of *t*Bu), 0.10 ppm (t, *J*=7.9 Hz, 4H; 2×CH_2_–Zn of *n*Bu); ^13^C{^1^H} NMR (100.62 MHz, 298 K, C_6_D_6_): *δ*=216.9 (metalated C of pyr), 151.5 (tertiary C of *t*Bu), 150.6 (aromatic C at *δ*_H_=8.89 ppm), 133.1 (aromatic C at *δ*_H_=8.11), 115.9 (aromatic C at *δ*_H_=6.70), 57.3 (2×CH_2_ PMDETA), 56.0 (2×CH_2_ PMDETA), 45.4 (4×CH_3_ PMDETA), 42.1 (1×CH_3_ PMDETA), 35.3 (2×CH_2_ *n*Bu), 34.2 (tertiary C *t*Bu), 31.7 (2×CH_2_ of *n*Bu), 30.8 (3×CH_3_ of *t*Bu), 16.2 (2×Zn–CH_2_ *n*Bu), 15.1 ppm (2×CH_3_ *n*Bu).

## Notes

Detailed facts of importance to specialist readers are published as ”Supporting Information”. Such documents are peer-reviewed, but not copy-edited or typeset. They are made available as submitted by the authors.

**Metal cooperation**: The direct zincation of various 4-substituted pyridines at the 2-position by potassium–dialkyl–TMP–zincate bases is manifested in novel dimeric dipotassium-capped anthracene-like structures with coordinatively saturated zinc centers (see figure).

## References

[[1]a] Mulvey RE (2006). Organometallics.

[b] Mulvey RE, Mongin F, Uchiyama M, Kondo Y (2007). Angew. Chem.

[c] Mulvey RE (2009). Acc. Chem. Res.

[[2]a] Barley HRL, Clegg W, Dale SH, Hevia E, Honeyman GW, Kennedy AR, Mulvey RE (2005). Angew. Chem.

[b] Andrikopoulos PC, Armstrong DR, Barley HRL, Clegg W, Dale SH, Hevia E, Honeyman GW, Kennedy AR, Mulvey RE (2005). J. Am. Chem. Soc.

[c] Clegg W, Dale SH, Hevia E, Honeyman GW, Mulvey RE (2006). Angew. Chem.

[d] Seggio A, Jutand A, Priem J, Mongin F (2008). Synlett.

[e] L’Helgoual’ch J-M, Seggio A, Chevallier F, Yonehara M, Jeanneau E, Uchiyama M, Mongin F (2008). J. Org. Chem.

[[3]a] Uchiyama M, Naka H, Matsumoto Y, Ohwada T (2004). J. Am. Chem. Soc.

[b] Garcia-Álvarez J, Graham DV, Kennedy AR, Mulvey RE, Weatherstone S (2006). Chem. Commun.

[c] Garcia-Álvarez J, Hevia E, Kennedy AR, Klett J, Mulvey RE (2007). Chem. Commun.

[d] Conway B, Hevia E, Garcia-Álvarez J, Graham DV, Kennedy AR, Mulvey RE (2007). Chem. Commun.

[e] Naka H, Uchiyama M, Matsumoto Y, Wheatley AEH, McPartlin M, Morey JV, Kondo Y (2007). J. Am. Chem. Soc.

[[4]a] L’Helgoual’ch J-M, Bentabed-Ababsa G, Chevallier F, Yonehara M, Uchiyama M, Derdour A, Mongin F (2008). Chem. Commun.

[b] L’Helgoual’ch J-M, Bentabed-Ababsa G, Chevallier F, Derdour A, Mongin F (2008). Synthesis.

[c] Bentabed-Ababsa G, Blanco F, Derdour A, Mongin F, Trecourt F, Queguiner G, Ballesteros R, Abarca B (2009). J. Org. Chem.

[5] Alborés P, Carrella LM, Clegg W, Garcia-Álvarez P, Kennedy AR, Klett J, Mulvey RE, Rentschler E, Russo L (2009). Angew. Chem.

[[6]a] Conway B, Hevia E, Kennedy AR, Mulvey RE (2007). Chem. Commun.

[b] Blair VL, Carrella LM, Clegg W, Conway B, Harrington RW, Hogg LM, Klett J, Mulvey RE, Rentschler E, Russo L (2008). Angew. Chem.

[c] Blair VL, Kennedy AR, Klett J, Mulvey RE (2008). Chem. Commun.

[d] Garcia-Álvarez P, Graham DV, Hevia E, Kennedy AR, Klett J, Mulvey RE, O’Hara CT, Weatherstone S (2008). Angew. Chem.

[[7]a] Carrella LM, Clegg W, Graham DV, Hogg LM, Kennedy AR, Klett J, Mulvey RE, Rentschler E, Russo L (2007). Angew. Chem.

[b] Blair VL, Clegg W, Conway B, Hevia E, Kennedy AR, Klett J, Mulvey RE, Russo L (2008). Chem. Eur. J.

[c] Blair VL, Carrella LM, Clegg W, Klett J, Mulvey RE, Rentschler E, Russo L (2009). Chem. Eur. J.

[[8]a] Kondo Y, Shilai M, Uchiyama M, Sakamoto T (1999). J. Am. Chem. Soc.

[b] Uchiyama M, Matsumoto Y, Nobuto D, Furuyama T, Yamaguchi K, Morokuma K (2006). J. Am. Chem. Soc.

[9] Dong Z, Clososki GC, Wunderlich SH, Unsinn A, Li J, Knochel P (2009). Chem. Eur. J.

[10] Conway B, Graham DV, Hevia E, Kennedy AR, Klett J, Mulvey RE (2008). Chem. Commun.

[11] Hart AJ, O’Brien DH, Russell CH (1974). J. Organomet. Chem.

[[12]a] Fabicon RM, Parvez M, Richey HG (1991). J. Am. Chem. Soc.

[b] Purdy AP, George CF (1992). Organometallics.

[c] Purdy AP, George CF (1994). Polyhedron.

[d] Rijnberg E, Boersma J, Jastrzebski JTBH, Lakin MT, Spek AL, van Koten G (1995). Chem. Commun.

[e] Rijnberg E, Boersma J, Jastrzebski JTBH, Lakin MT, Spek AL, van Koten G (1997). Organometallics.

[f] Rijnberg E, Richter B, Thiele K-H, Boersma J, Veldman N, Spek AL, van Koten G (1998). Inorg. Chem.

[g] Darensbourg DJ, Niezgoda SA, Draper JD, Reibenspies JH (1999). Inorg. Chem.

[h] Forbes GC, Kennedy AR, Mulvey RE, Roberts BA, Rowlings RB (2002). Organometallics.

[i] Clegg W, Forbes GC, Kennedy AR, Mulvey RE, Liddle ST (2003). Chem. Commun.

[j] Merz K, Block S, Schoenen R, Driess M (2003). Dalton Trans.

[k] Cremer U, Pantenburg I, Ruschewitz U (2003). Inorg. Chem.

[l] Baillie SE, Hevia E, Kennedy AR, Mulvey RE (2007). Organometallics.

[m] Gren CK, Hanusa TP, Rheingold AL (2007). Organometallics.

[n] Alvarez E, Grirrane A, Resa I, del Rio D, Rodríguez A, Carmona E (2007). Angew. Chem.

[13] Garcia F, Hopkins A, Kowenicki RA, McPartlin M, Silvia JS, Rawson JM, Rodgers MC, Wright DS (2007). Chem. Commun.

[14] Nakatsu K, Kinoshitu K, Kanda H, Isobe K, Nakamura Y, Kawaguchi S (1980). Chem. Lett.

[15] Churakov AV, Krut′ko DP, Borzov MV, Kirsanov RS, Belov SA, Howard JAK (2006). Acta Crystallogr. Sect. E.

[16] Schulz S, Thomas F, Priesmann WM, Nieger M (2006). Organometallics.

[17] Engering J, Jansen M (2003). Z. Anorg. Allg. Chem.

[18] Schlosser M, Mongin F (2007). Chem. Soc. Rev.

[19] Anders E, Opitz A, Bauer W (1991). Synthesis.

[20] Brown HC, Murphey WA (1951). J. Am. Chem. Soc.

[21] Stalick WM, Pines H (1970). J. Org. Chem.

[22] Gros P, Fort Y (2002). J. Org. Chem.

[23] Nobuto D, Uchiyama M (2008). J. Org. Chem.

[24] Uchiyama M, Matsumoto Y, Usui S, Hashimoto Y, Morokuma K Angew. Chem.

[25] Kondo Y, Morey JV, Morgan JC, Naka H, Nobuto D, Raithby PR, Uchiyama M, Wheatley AEH (2007). J. Am. Chem. Soc.

[26] Clegg W, Conway B, Hevia E, McCall MD, Russo L, Mulvey RE (2009). J. Am. Chem. Soc.

[27] Barnett NDR, Clegg W, Kennedy AR, Mulvey RE, Weatherstone S (2005). Chem. Commun.

[28] Naka H, Morey JV, Haywood J, Eisler DJ, McPartlin M, Garcia F, Kudo H, Kondo Y, Uchiyama M, Wheatley AEH (2008). J. Am. Chem. Soc.

[29] Furuyama T, Yonehara M, Arimoto S, Kobayashi M, Matsumoto Y, Uchiyama M (2008). Chem. Eur. J.

[30] Hlavinka ML, Greco JF, Hagadorn JR (2005). Chem. Commun.

[31] Andrikopoulos PC, Armstrong DR, Hevia E, Kennedy AR, Mulvey RE, O’Hara CT (2005). Chem. Commun.

[32] COLLECT (2000). Program for Data Collection.

[33] Sheldrick GM (2008). Acta Crystallogr. Sect. A.

