# Supplementary material for: Structurally Defined Potassium-Mediated Zincation of Pyridine and 4-R-Substituted Pyridines (R=Et, iPr, tBu, Ph, and Me2N) by Using Dialkyl–TMP–Zincate Bases
Source: Chemistry. 2009 Jun 16;15(29):7074–82. doi: 10.1002/chem.200900549 (PMC3784044; doi:10.1002/chem.200900549)
Supplement: Supplementary file 1 [file chem0015-7074-SD1.pdf]

CHEMISTRY
A EUROPEAN JOURNAL

Supporting Information

© Copyright Wiley-VCH Verlag GmbH & Co. KGaA, 69451 Weinheim, 2009

Structurally-defined potassium-mediated zincation of pyridine and 4-R-substituted pyridines (R = Et, *i*Pr, *t*Bu, Ph, Me_2N) using bisalkyl TMP-zincate bases

William Clegg,[b] Ben Conway,*[a] David V. Graham,[a] Eva Hevia,[a] Alan R. Kennedy,[a] Robert E. Mulvey,*[a] Luca Russo,[b] and Dominic S. Wright [c]

[a] B. Conway, D. V. Graham, Dr. E. Hevia, Dr. A. R. Kennedy, Prof. R. E. Mulvey
WestCHEM, Department of Pure and Applied Chemistry
University of Strathclyde, Glasgow, G1 1XL (UK)
Fax: (+44) 141-552-0876
E-mail: benjamin.conway@strath.ac.uk

[b] Prof. W. Clegg, Dr. L. Russo
School of Chemistry
Newcastle University, Newcastle upon Tyne, NE1 7RU (UK)

[c] Dr. D. S. Wright
Chemistry Department
University of Cambridge, Lensfield Road, Cambridge, CB2 1EW (UK)

Figure 1: Molecular structure of the heterobimetallic base [PMDETA.K(TMP)(Et)Zn(Et)] **1**. Minor disorder and hydrogen atoms are omitted for clarity.

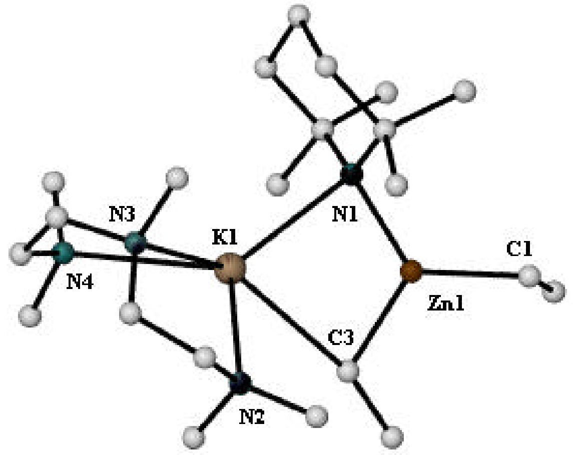

Figure 2: Molecular structure of the heterobimetallic base [PMDETA.K(TMP)(*n*Bu)Zn(*n*Bu)] **2**. Minor disorder and hydrogen atoms are omitted for clarity.

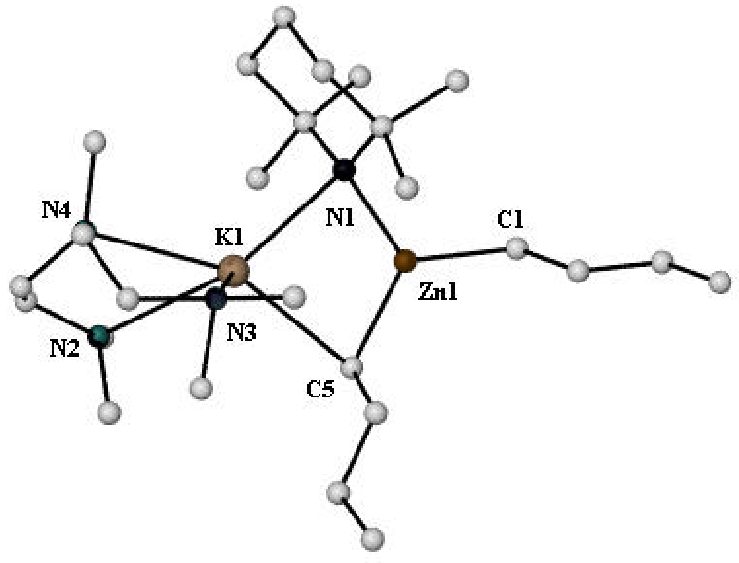

Figure 3: Molecular structure of the 2-zincated DMAP derivative [{PMDETA.K[2-Zn(Et)$_2$-4-Me$_2$N-C$_5$H$_3$N]}$_2$] **3.** Hydrogen atoms are omitted for clarity.

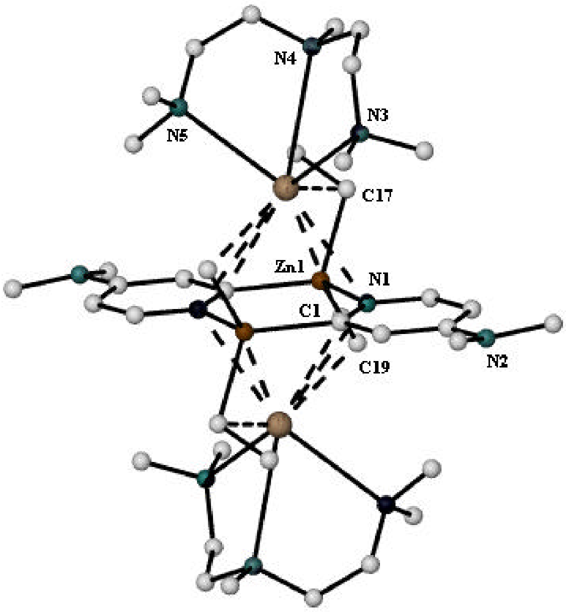

Figure 4: Molecular structure of the minor (yield, 7%) 2-zincated 4-ethylpyridine product [{PMDETA.K[2-Zn(Et)$_2$-4-Et-C_5H_3N]}$_2$] **5.** Minor disorder and hydrogen atoms are omitted for clarity.

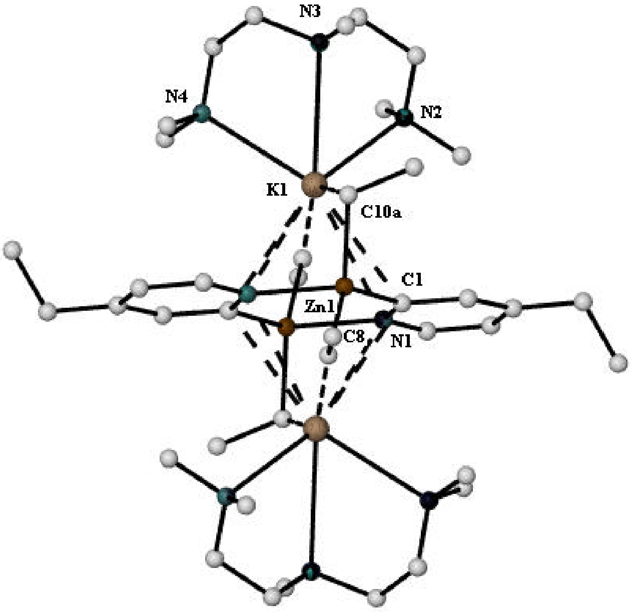

Figure 5: Molecular structure of the 2-zincated 4-isopropylpyridine complex [{PMDETA.K[2-Zn(Et)$_2$-4-^{i}Pr-C_5H_3N]}$_2$] **6.** Hydrogen atoms are omitted for clarity.

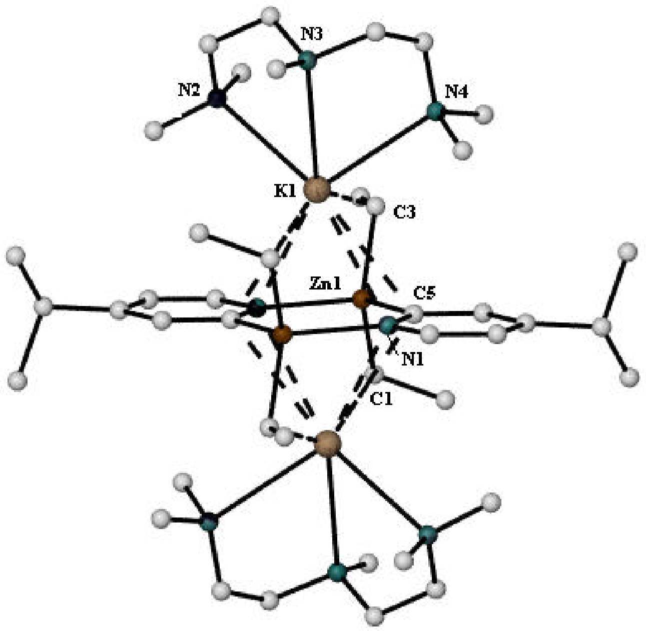

Figure 6: Molecular structure of the 2-zincated 4-tert-butylpyridine product [{PMDETA.K[2-Zn(Et)$_2$-4-^{t}Bu-C_5H_3N]}$_2$] **7.** Minor disorder and hydrogen atoms are omitted for clarity.

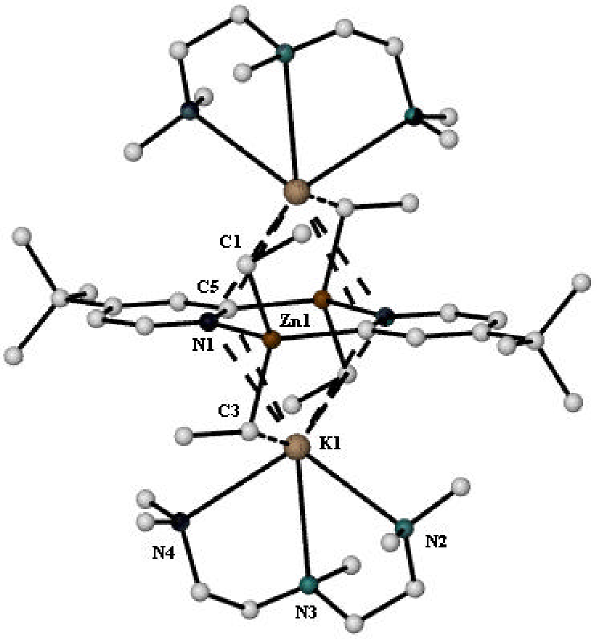

Figure 7: Molecular structure of one of the two independent molecules in the unit cell of **8**, [{PMDETA.K[2-Zn(Et)$_2$-4-Ph-C_5H_3N]}$_2$]. Minor disorder and hydrogen atoms are omitted for clarity.

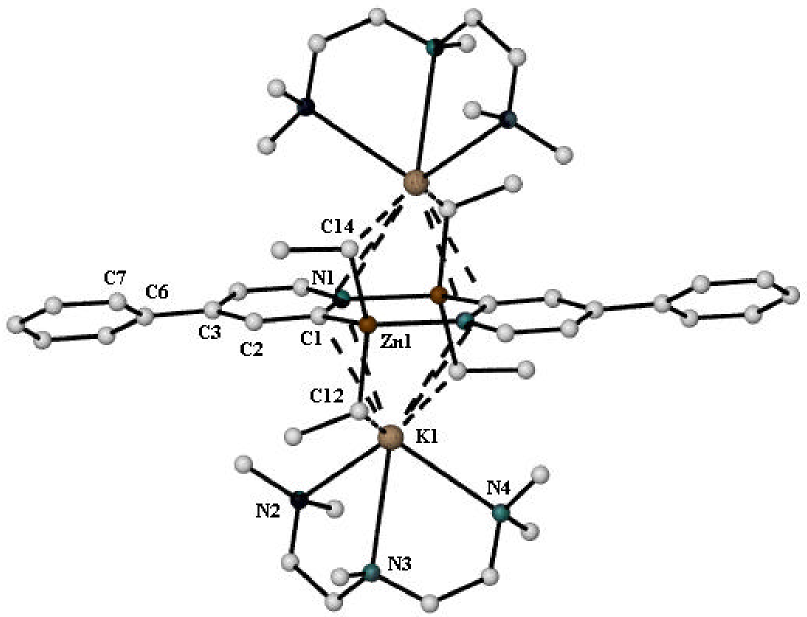

Stoichiometric and catalytic TMPH experimental results:

General Points:

Each reaction was conducted on a 2 mmol scale, in the same volume of dried hexane (10 mL) at room temperature under an argon atmosphere.

Each time entry in the tables below represents a 0.5 mL aliquot taken from the reaction mixture. This aliquot was transferred to a second Schlenk tube, the solvent removed *in vacuo* and the residue was dissolved in 0.5 mL of deuterated benzene.

The % conversion of 4-*t*-butylpyridine to the 2-zincated 4-*t*-butylpyridine product was calculated using the integrations of the metalated 4-*t*-butylpyridine and the free 4-*t*-butylpyridine resonances from the ^{1}H NMR spectrum.

Et_2Zn reactions

Reaction: KCH_2SiMe_3 + PMDETA + TMPH + Et_2Zn + 4-*t*-butylpyridine

Time (hrs)	conversion %
0	0
0.03	53
1	86

Reaction: KCH_2SiMe_3 + PMDETA + Et_2Zn + TMPH + 4-*t*-butylpyridine

Time (hrs)	conversion %
0	0
0.03	0
1	0
22.5	42
27	49
95.5	77

Reaction: KCH_2SiMe_3 + PMDETA + Et_2Zn + 0.1 TMPH + 4-*t*-butylpyridine

Time (hrs)	conversion %
0	0
18	6
42	15
66	25
90	32
186	49

Reaction: KCH_2SiMe_3 + PMDETA + Et_2Zn + 4-*t*-butylpyridine

Time (hrs)	conversion %
0	0
18	0
42	0
66	0
90	0
186	4

***n*Bu$_2$Zn reactions**

Reaction: KCH_2SiMe_3 + PMDETA + TMPH + *n*Bu_2Zn + 4-*t*-butylpyridine

Time (hrs)	conversion %
0	0
0.03	96

Reaction: KCH_2SiMe_3 + PMDETA + *n*Bu_2Zn + TMPH + 4-*t*-butylpyridine

Time (hrs)	conversion %
0	0
0.03	0
1	27
19	86

Reaction: KCH_2SiMe_3 + PMDETA + *n*Bu_2Zn + 0.1 TMPH + 4-*t*-butylpyridine

Time (hrs)	conversion %
0	0
0.03	0
1.25	0
67	55
95.5	79
114.5	85

Reaction: KCH_2SiMe_3 + PMDETA + *n*Bu_2Zn + 0.5 TMPH + 4-*t*-butylpyridine

Time (hrs)	conversion %
0	0
0.03	0
1	3
17	50
24	81
41	87

Reaction: KCH_2SiMe_3 + PMDETA + *n*Bu_2Zn + 4-*t*-butylpyridine

Time (hrs)	conversion %
0	0
22	0
66	8
95	11
164	16

Crystallography Experimental.

All data were collected at low temperature using Mo Ka radiation (? = 0.71073 Å). Compounds **5** and **8** were measured on an Oxford Diffraction Gemini S instrument and all others on Nonius Kappa CCD diffractometers. All structures were solved and refined using the SHELX range of programmes. Full details for each individual structure are given in the deposited cif files.

Compound	**1**	**2**	**3**	**4**	**5**	**6**	**7**	**8**	**9**
Formula	$C_{22}H_{51}KN_4Zn$	$C_{26}H_{59}KN_4Zn$	$C_{40}H_{84}K_2N_{10}Zn_2$	$C_{36}H_{74}K_2N_8Zn_2$	$C_{40}H_{82}K_2N_8Zn_2$	$C_{42}H_{86}K_2N_8Zn_2$	$C_{44}H_{90}K_2N_8Zn_2$	$C_{48}H_{82}K_2N_8Zn_2$	$C_{48}H_{82}K_2N_8Zn_2$
F. weight	476.14	532.24	914.11	827.97	884.08	915.15	940.18	980.16	1052.39
Cryst syst.	orthorhombic	triclinic	monoclinic	monoclinic	monoclinic	monoclinic	monoclinic	triclinic	monoclinic
sp. gr.	$P2_12_12_1$	P-1	C2/c	$P2_1/n$	$P2_1/n$	$P2_1/c$	$P2_1/n$	P-1	C2/c
a Å	8.0619(2)	10.5721(2)	28.3709(8)	11.436(2)	13.4732(3)	13.4980(5)	13.441(3)	9.8074(6)	27.500(7)
b Å	15.9886(4)	10.7949(3)	12.1545(3)	16.493(3)	12.6443(3)	13.1963(4)	12.970(3)	13.1617(9)	12.8693(10)
c Å	21.2669(6)	15.6143(4)	18.5001(5)	12.218(2)	14.5974(3)	18.1640(5)	15.234(3)	22.4372(15)	20.695(3)
α °	90	86.851(2)	90	90	90	90	90	86.341(5)	90
β °	90	78.881(2)	128.617(1)	97.27(3)	97.405(2)	128.224(2)	92.67(2)	88.833(5)	123.765(14)
γ °	90	64.329(2)	90	90	90	90	90	69.464(6)	90
V $Å^3$	2741.27(12)	1575.12(7)	4984.5(2)	2286.0(8)	2466.06(9)	2541.75(14)	2652.7(9)	2706.7(3)	6088.7(18)
Z	4	2	4	2	2	2	2	2	4
μ mm^{-1}	1.061	0.930	1.218	1.263	1.175	1.142	1.096	1.077	0.962
Temp. K	123	150	123	150	123	123	150	123	150
refls. coll.		10981	10886	15618	29148	9756	15975	37589	17368
refls. uniq.	4780	5500	5679	3984	6670	5809	4618	11451	5223
Rint		0.0154	0.0215	0.0838	0.0247	0.0218	0.0263	0.0533	0.1214
reflns. obs.	4132	4669	4762	2384	4559	4405	3875	6312	3764
GoF	1.121	1.043	1.062	1.020	1.044	1.088	1.097	0.872	1.015
R[I>2σ(I)]	0.0764	0.0270	0.0274	0.0640	0.0351	0.0516	0.0247	0.0455	0.1077
Rw2	0.1607	0.0700	0.0672	0.0964	0.0888	0.1335	0.0596	0.1121	0.2390

CCDC 683512 (**1**), 721902 (**2**), 683513 (**3**), 721903 (**4**), 721904 (**5**), 721905 (**6**), 721906 (**7**), 721907 (**8**) and 721908 (**9**) contain the supplementary crystallographic data for this paper. This data can be obtained free of charge from The Cambridge Crystallographic Data Centre via www.ccdc.cam.ac.uk/data_request/cif.
